# MiR-181b suppress glioblastoma multiforme growth through inhibition of SP1-mediated glucose metabolism

**DOI:** 10.1186/s12935-020-1149-7

**Published:** 2020-03-04

**Authors:** JianXing Yin, ZhuMei Shi, WenJin Wei, Chenfei Lu, Yutian Wei, Wei Yan, Rui Li, JunXia Zhang, YongPing You, XieFeng Wang

**Affiliations:** 1 Department of Neurosurgery, The First Affiliated Hospital of Nanjing Medical University, Nanjing, China; 2 Department of Neurosurgery, The Affiliated Ganzhou Hospital of Nanchang University, 16 Meiguan Avenue, Ganzhou, 341000 Jiangxi China

**Keywords:** miR-181b, SP1, Glucose metabolism, Glioma

## Abstract

**Background:**

Glucose metabolic reprogramming is a significant hallmark of malignant tumors including GBM. Previous studies suggest that microRNAs play key roles in modulating this process in GBM cells. miR-181b acts as a tumor suppressor miRNA in influencing glioma tumorigenesis. Our previous results showed that miR-181b was down-regulated in glioma cells and tissues.

**Methods:**

The extracellular acidification rate (ECAR), colony formation assay and levels of Glut1 and PKM2 were measured to assess the glucose metabolic and proliferation changes in GBM cells overexpressing miR-181b. Immunoblotting and luciferase reporter assay were performed to confirm the expression and role of SP1 as a direct target of miR-181b. ChIP assay was used to figure out the transcriptional regulation of SP1 on Glut1 and PKM2. In vivo study was examined for the role of miR-181b in GBM cells.

**Results:**

MiR-181b overexpression significantly reduced the glucose metabolic and colony formation ability of GBM cells. And, SP1 was confirmed as a direct target of miR-181b while upregulation of SP1 could reverse the influence of overexpression of miR-181b. Furthermore, Glut1 and PKM2 could be regulated by SP1. Finally, miR-181b could inhibit the tumor growth in vivo.

**Conclusions:**

Our article demonstrated the inhibitory effect of miR-181b on glucose metabolism and proliferation in GBM by suppressing SP1 expression.

## Background

Glioma is the most common primary brain tumor in adults [1]. Glioblastoma multiforme (GBM), which is categorised as a WHO IV tumor, accounts for about half of glioma classification [2]. Current treatment including surgical resection and followed by radio/chemotherapy have largely improved the median survival time [3, 4]. However, due to the aggressive hallmarks such as: sustaining proliferation, acquired chemo-resistance and deregulating glucose metabolism, the prognosis for GBM remains poor [5, 6].

The changed glucose metabolism has been listed as an important accomplice that directly contributes to carcinogenesis [7, 8]. Warburg effect (aerobic glycolysis) represents major metabolic phenotypes for energy production. Under aerobic conditions, lactate was produced from glucose metabolism in tumors cells, while normal differentiated cells extract energy via oxidative-phosphorylating glucose. Glioma also exhibits the Warburg effect. It was reported that glycolytic metabolism of tumor tissues is three times higher than normal brain tissues [9]. Multiple signaling pathways are linked to this process, such as c-MYC and HIF-1 regulatory network [10]. However, there is still much need to investigate about how glucose metabolism in glioma is regulated.

Specificity protein 1 (SP1), a member of Sp/Kruppel-like factor (KLF) transcription factors family, is one of the firstly identified eukaryotic transactivators [11]. Some literature has reported the expression of SP1 was dysregulated in various types of cancers including glioma [12–14]. Overexpression of SP1 plays an important role in regulating multiple vital oncoproteins, such as EGFR and VEGF [15]. And Overexpression of SP1 is associated with poor clinical outcome [16, 17].

Aberrant expression of miR-181 family has been found in many tumors. Our group firstly identified that miR-181a and miR-181b were down-regulation in human glioma tissues and cells, which played a critical role in the pathogenesis of gliomas. For example, our previous work revealed that miR-181b modulates chemo-sensitivity of GBM cells to TMZ via targeting EGFR [18]. In this research, we tend to discover more mechanism of miR-181b on effecting GBM glucose metabolism.

## Materials and methods

### Cell culture

Two human GBM cell lines (U87 and U251) were purchased from the American Type Culture Collection (ATCC, USA) and maintained in Dulbecco’s modified Eagle medium (DMEM, Gibco, USA) containing 10% fetal bovine serum (FBS, Gibco, USA) 1% penicillin and streptomycin. All cells were cultured at 37 °C in humidified atmosphere with 5% CO_2_.

### Clinical samples

A total of 20 clinical samples (5 normal brain specimens, 5 WHO grade II, 5 WHO grade III and 5 WHO grade IV glioma specimens) were obtained from the Department of Neurosurgery at the First Affiliated Hospital of NanJing Medical University and stored up in liquid nitrogen immediately after surgical resection.

### Lentiviral packaging and stable cell line establishment

U87 and U251 cells that stably overexpressing miR-181b or its negative control (miR-NC) were constructed before for our previous research [18].

### Reverse transcription-quantitative PCR

Total RNA was extracted from cells or tissue samples using TRIzol^®^ Reagent (Invitrogen). A stem loop-specific primer method was performed to measure miR-181b expression [19]. QRT-PCR was performed using the Applied Biosystems 7500 Sequence Detection System (Thermo Fisher Scientific, USA) following the manufacturer’s instructions. And the relative expression of miR-181b and SP1 fold changes were calculated by relative quantification (2^−△△CT^) normalized to β-actin or U6.

### Immunoblotting

Protein extraction and immunoblotting analysis were performed as described previously [20]. The following antibodies were used: SP1 (1:1000, Cell Signaling Technology, USA), PKM2 (1:1000, Cell Signaling Technology, USA), Glut1 (1:1000, abcam, UK) and Actin (1:1000, Abcam, UK).

### Extracellular acidification rate

Seahorse XF 96 Extracellular Flux Analyzer (Agilent Technologies, Santa Clara, CA, USA) and Seahorse XF Glycolysis Stress Test Kit were performed to measure the extracellular acidification rate (ECAR), 1 × 10^4^ cells were incubated overnight in the Seahorse XF 96 cell culture microplate. Then, the assay medium (pH = 7.4) which unbuffered DMEM with 2 mM l-glutamine was used to wash the cells and go on incubating the cells for an hour in a 37 °C incubator without CO_2_. After that, the microplates were loaded into the Seahorse Analyzer for 3 cycles according to the manufacturer’s instructions. At the specified time, we injected 10 mM glucose, 1.0 μM oxidative phosphorylation inhibitor oligomycin, and 50 mM glycolytic inhibitor 2-deoxyglucose (2-DG), into each well for ECAR measurement After the assays, cells were fixed, stained (1% crystal violet) and counted. All detected values were normalized to the cell number. Calculated as follows: Glycolysis rate = (Maximum rate measurement before oligomycin injection) − (Last rate measurement before glucose injection). Glycolytic capacity = (Maximum rate measurement after oligomycin injection) − (Last rate measurement before glucose injection). ECAR is presented in mpH/min.

### Colony formation assay

Cells in the logarithmic phase of growth were seeded in six-well plates (200 cells/well). Approximately 14 days later, colonies were observed and fixed by methanol and stained (1% crystal violet) (Sigma, USA). One visible colony counted 450 cells.

### Luciferase reporter assay

A luciferase reporter assay was used to detect the binding of miR-181b to SP1. Briefly, the SP1 3′-UTR are cloned into the SacI and HindIII sites of the pmiRNA-Report vector (Genechem, Shanghai, China) and confirmed by a sequencing to form the wild-type (WT) and mutated (Mut). Cells, which co-transfected with the WT or Mut reporter plasmid, pRLTK plasmid, and the miR-181b mimic or miR-NC were seeded in the 24-well plate. After transfection 24 h, promega Dual Luciferase Reporter Assay System (Promega) was performed to measure the luciferase activity.

To identify the regulation of SP1 on PKM2 and Glut1 promoter region, U87 and U251 cells were seeded in 24-well plate at a density of 6 × 10^4^ cells per well for 24 h before transfection. The cells were co-transfected with a mixture of luciferase reporter vectors (pGL3-basic) containing wild type sequence or mutant sequence of specific PKM2 and Glut1 promoter fragment. After 48 h, the luciferase activity was measured using a dual luciferase reporter assay system (Promega, Madison, WI, USA) according to the manufacturer’s protocol.

### Chromatin immunoprecipitation assay

Chromatin immunoprecipitation (ChIP) assays were employed using ChIP Assay Kits (Abcam) according to the manufacturer’s instructions. Briefly, U87 and U251 chromatin were incubated with Anti-SP1. Purified immunoprecipitated DNA was prepared for the PCR with primers (Forward: TCTGCAGGATTCCAGACCCT; Reverse: TGTTAGGACCGCGGAACAAG) for the promoter of PKM2 or with primers (Forward: CTTGAGCCCAGGAGTTTGAG; Reverse: GCCGAGGCTGTCTTCTTATG) for the promoter of Glut1.

### Orthotopic xenograft studies

6 weeks old female BALB/c nude mice were purchased from the Shanghai Experimental Animal Center (Shanghai, China). 15 mice were divided into three groups randomly. 5 × 10^6^ U87 cells which stably transfected with luciferase virus in 100 μl were injected intracranially into mice (NC group). miR-181b-overexpressing U87 cells (miR-181b group) and miR-181b plus SP1-overexpressing U87 cells (miR-181b + SP1 group) were similarly injected. Luminescence imaging (IVIS Spectrum, PerkinElmer, USA) was used to measure the tumor volume each week.

### Statistical analysis

All results were performed at least three independent repeats of the experiments. GraphPad 5.0 software was used to analyze the data. *P *< 0.05 was considered to be statistically significant.

## Results

### MiR-181b suppresses the glucose metabolism and proliferation in GBM cells

**Fig. 1:**
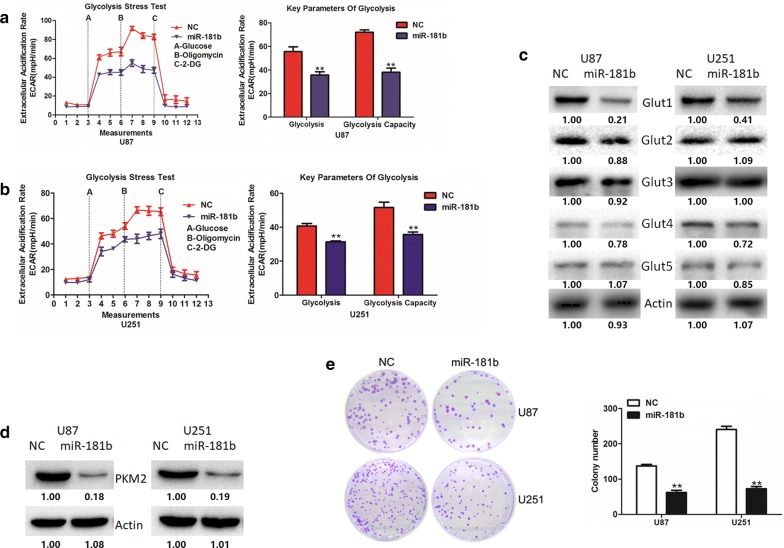
miR-181b overexpression inhibits glioma cells aerobic glycolysis and proliferation. **a**, **b** ECAR was measured through the Glycolysis Stress in U87 and U251 cells after the cells were transfected miR-NC or miR-181b. **c** The expression of Glut1-5 were measured by Western blot in miR-NC or miR-181b-transfected U87 and U251 cells. **d** The expression of PKM2 were measured by Western blot in miR-NC or miR-181b-transfected U87 and U251 cells. **e** Colony formation ability of miR-NC or miR-181b-transfected U87 and U251 cells. *P < 0.05, **P < 0.01. All results are representative of at least three independent experiments

In our previous results, we confirmed that miR-181b was down-regulated in human GBM cells and tissues [18]. And miR-181b could act as a tumor suppressor gene, providing new therapeutic target for GBM treatment. In this article, we tend to further explore the function of miR-181b on glucose metabolism and tumor growth in GBM. Firstly, we investigate the role of miR-181b in GBM cells glucose metabolism and ECAR was measured to assess the glycolysis rate. As shown in Fig. 1a, b, glycolysis rate and glycolytic capacity were significantly reduced in miR-181b-overexpressed group of U87 and U251 cells.

As we all know, glucose transporters are the most important regulators of glycolysis [21]. Therefore, we firstly detected glucose transporters expression levels after overexpressing miR-181b. And the results showed that glut1 was mostly suppressed among the 5 glucose transporters (Fig. 1c). Meanwhile, our previous study identified that PKM2 promotes glucose metabolism and cell growth in gliomas [22]. Therefore, to extend this study, we also detected PKM2 expression levels, and PKM2 was significantly suppressed by miR-181b (Fig. 1d). Next, we employed colony formation assays to explore the function of miR-181b on proliferation in GBM cells. Meanwhile, miR-181b inhibited the cell proliferation of U87 and U251 (Fig. 1e). These results indicated that miR-181b might regulate glucose metabolism in glioma cells and thus affect cell proliferation.

### SP1 is a direct target of miR-181b

**Fig. 2:**
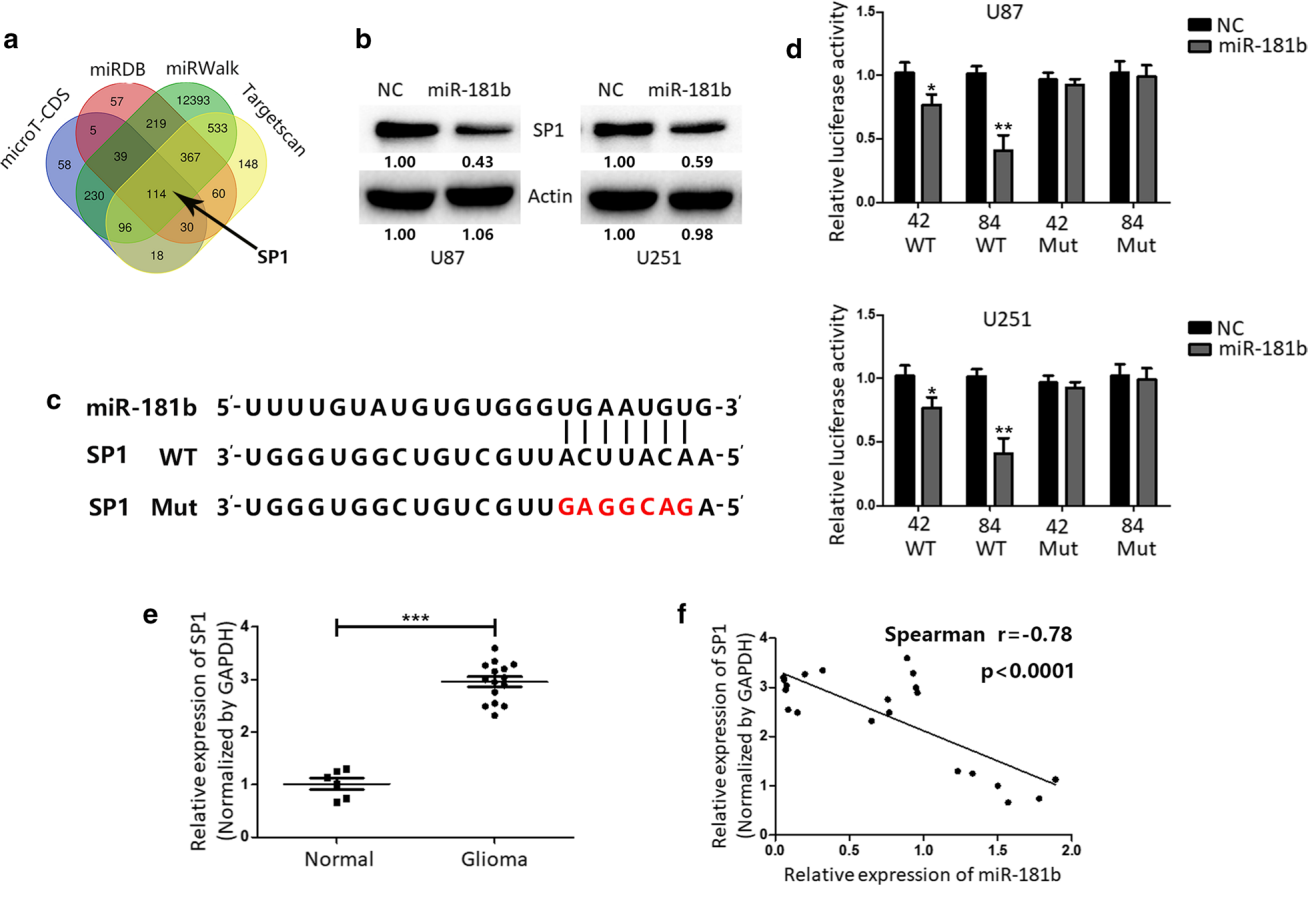
miR-181b directly targets SP1 in GBM cells. **a** Venn diagram displaying miR-181b computationally predicted to target SP1 by four different prediction algorithms. **b** The expression of SP1 were measured by Western blot in miR-NC or miR-181b-transfected U87 and U251 cells. **c** Predicted miR-181b target sequence in 3′ UTR of SP1. And target sequences were mutated. **d** Luciferase assays of U87 and U251 cells transfected with SP1-3′-UTRs-WT or SP1-3′-UTRs-Mut reporter together with miR-NC or miR-181b. **e** SP1 expression levels in six normal brain tissues and 15 glioma specimens were measured by RT-PCR. **f** Spearman correlation analysis was used to evaluate the correlations between miR-181b and SP1 in both normal brain tissues and glioma specimens. *P < 0.05, **P < 0.01. All results are representative of at least three independent experiments

As we all known that miRNAs could repress transcription by binding to complementary sequences in the 3′-UTRs. Bioinformatics analysis (microT-CDS, miRDB, miRWalk and Targetscan) was used in order to explore the potential molecular mechanisms miR-181b. The results showed that SP1 might be a potential target of miR-181b (Fig. 2a). And, we found that the SP1 expression level was dramatically down-regulation after miR-181b overexpression (Fig. 2b). To further confirm the hypothesis, luciferase reporter assay was performed to identificate whether miR-181b could directly bind to the 3′-UTR of SP1. As showed in Fig. 2c, d, the luciferase activity in U251 and U87 cells were significantly reduced after transfection with WT 3′-UTR of SP1 and miR-181b mimics but not the mutant vector. In addition, we examined the SP1 levels in normal and glioma tissues and the results indicated a negative relationship between miR-181b and SP1 (Fig. 2e, f). Together, these data suggest that miR-181b directly regulate SP1.

### SP1 down-regulation has a similar effect with miR-181b over-expression in U87 and U251 cells

**Fig. 3:**
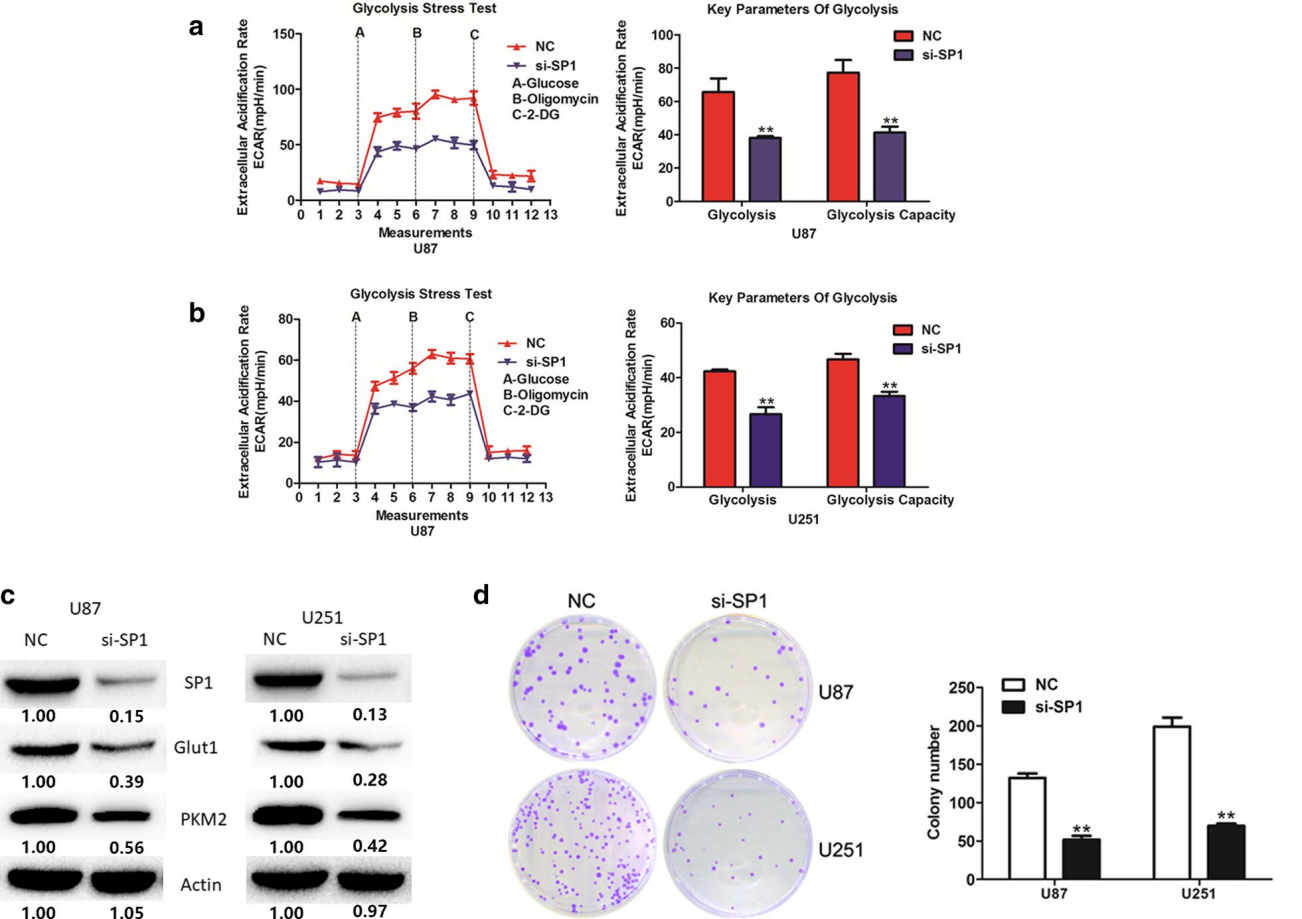
SP1 promotes glioma cells aerobic glycolysis and proliferation. **a**, **b** ECAR were measured through the Glycolysis Stress in U87 and U251 cells after the cells were transfected si-NC or si-SP1. **c** The expression of SP1, Glut1 and PKM2 were measured by Western blot in si-NC or si-SP1-transfected U87 and U251 cells. **d** Colony formation ability of si-NC or si-SP1-transfected U87 and U251 cells. *P < 0.05, **P < 0.01. All results are representative of at least three independent experiments

Since we have confirmed that SP1 was a direct target of miR-181b, we tend to investigate whether SP1 could affect the glucose metabolism and proliferation in GBM cells. To verify the question, siNC or si-SP1 was transfected into glioma cells. Immunoblotting assay was used to confirm the knock-down effectively (Fig. 3c). Extracellular acidification assays, Immunoblotting assay and colony formation were employed to evaluate the effects of SP1 knockdown on cell glucose metabolism and proliferation in U87 and U251 cells. As expected, knock-down of SP1 expression inhibited the glucose metabolism and cells proliferation (Fig. 3a–d). These results showed that SP1 down-regulation consistent with the impact of miR-181b over-expression in GBM cells.

### MiR-181b suppresses the glucose metabolism and cell proliferation by directly targeting SP1 in GBM cells

**Fig. 4:**
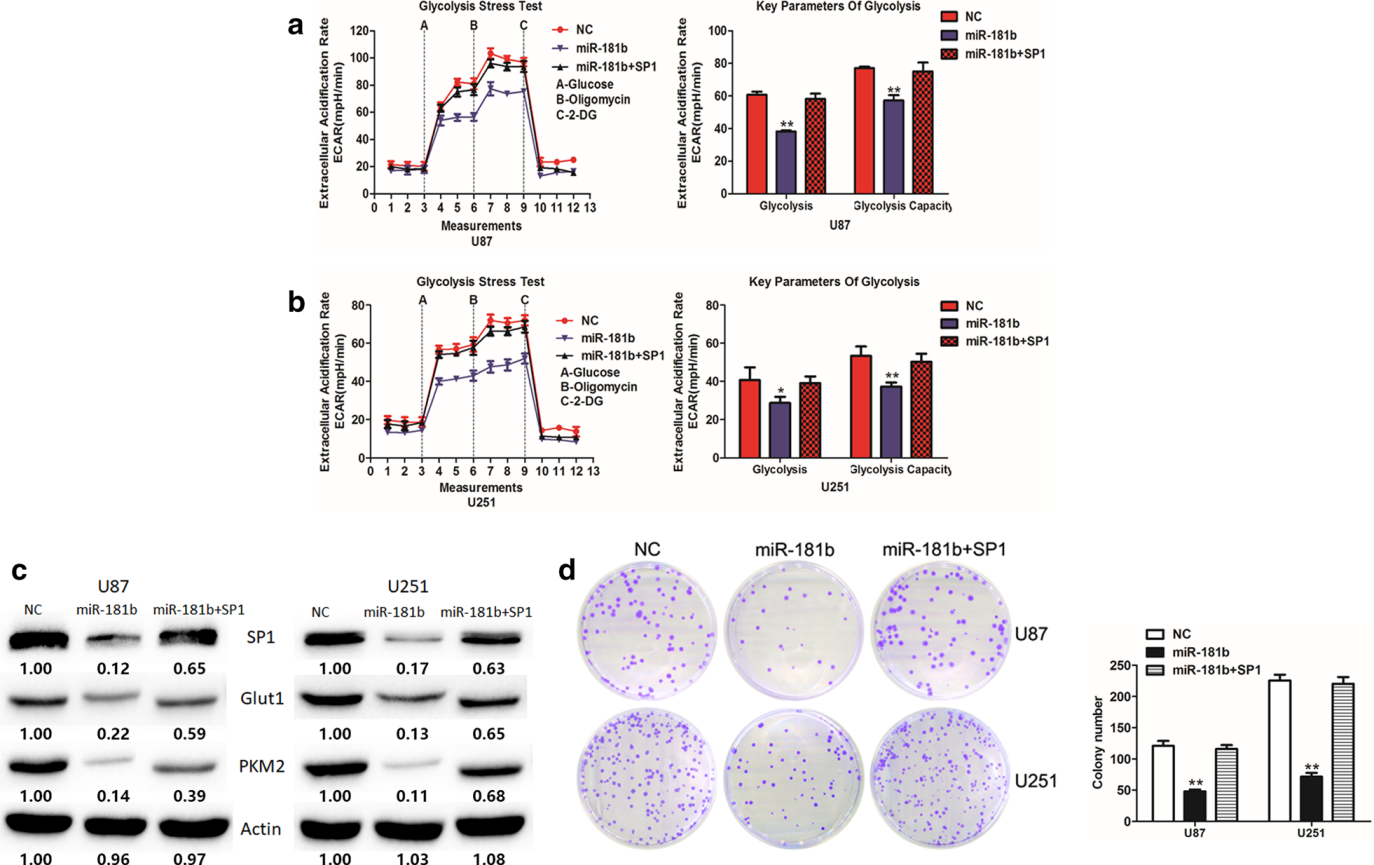
miR-181b overexpression inhibits glioma cells aerobic glycolysis and proliferation by targeting SP1. **a**, **b** ECAR were measured through the Glycolysis Stress in U87 and U251 cells after the cells were transfected miR-NC or miR-181b together with or without transfected with SP1 plasmid. **c** The expression of SP1, Glut1 and PKM2 were measured by Western blot in in U87 and U251 cells after the cells were transfected miR-NC or miR-181b together with or without transfected with SP1 plasmid. **d** Colony formation ability of U87 and U251 cells after the cells were transfected miR-NC or miR-181b together with or without transfected with SP1 plasmid. *P < 0.05, **P < 0.01. All results are representative of at least three independent experiments

In the above sections, we have verified SP1 is a direct target of miR-181b, and investigated the biological function of miR-181b and SP1 on glucose metabolism and cell proliferation in GBM cells. Therefore, we addressed whether miR-181b suppresses GBM progression by targeting SP1. U87 and U251 cells which stably over-expressing miR-181b or miR-NC were transfected with pcDNA3-SP1 plasmids. The decreased levels of SP1 due to miR-181b over-expression were partly reversed by SP1 over-expression (Fig. 4c). As well as SP1, Glut1 and PKM2 have the same changes. To further confirm, SP1 is a vital target of miR-181b in glucose metabolism and cell proliferation. SP1 over-expression rescued the inhibition of glucose metabolism and cell proliferation which induced by miR-181b (Fig. 4a, b and d). Overall, the above results suggest that SP1 is a functional target of miR-181b in GBM cells.

### SP1 modulates glucose metabolism and cell proliferation by targeting Glut1 and PKM2 in GBM

**Fig. 5:**
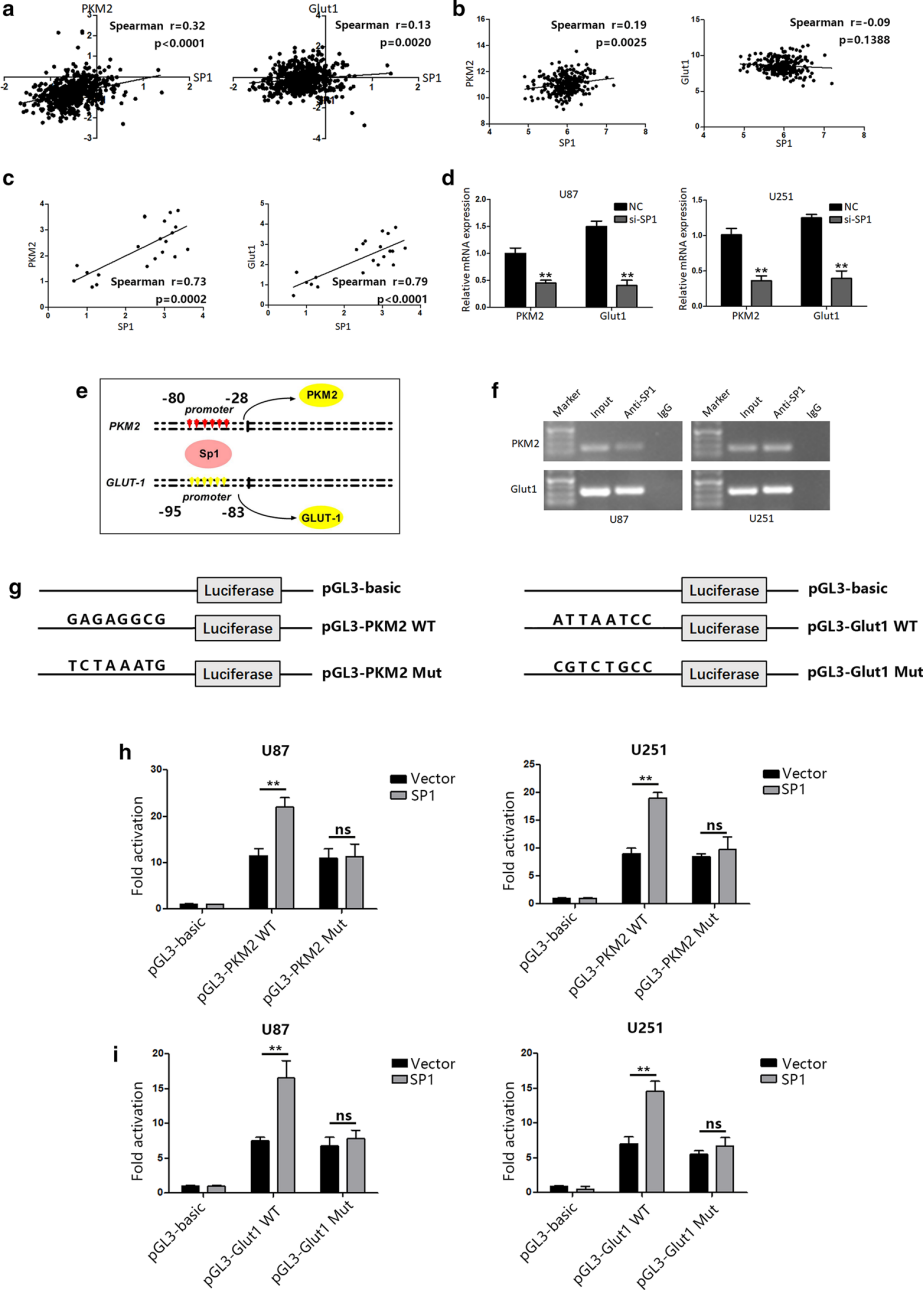
SP1 transcriptionally regulates Glut1 and PKM2. **a** Spearman correlation analysis of SP1 expression with PKM2 expression and Glut1 expression, as indicated by the TCGA data sets. **b** Spearman correlation analysis of SP1 expression with PKM2 expression and Glut1 expression, as indicated by the CGGA data sets. **c** Spearman correlation analysis of SP1 expression with PKM2 expression and Glut1 expression in both normal brain tissues and glioma specimens. **d** The mRNA level of Glut1 and PKM2 were measured by RT-PCR in si-NC or si-SP1-transfected U87 and U251 cells. **e** Schematic representation of the transcriptionally regulation of SP1 on Glut1 and PKM2. **f** Chromatin immunoprecipitation PCR products for putative SP1-binding sites. **g** Schematic representation of the PKM2 and Glut1 promoter reporters. **h** The luciferase reporter assays were used to detect the PKM2 promoter reporter activity in U87 and U251 cells transfected with SP1-overexpressing plasmid or its corresponding control. **i** The luciferase reporter assays were used to detect the Glut1 promoter reporter activity in U87 and U251 cells transfected with SP1-overexpressing plasmid or its corresponding control. *P < 0.05, **P < 0.01. All results are representative of at least three independent experiments

Above results showed that Glut1 and PKM2 levels were significant affected by SP1 levels. And, the correlation analyses of SP1 and Glut1 or PKM2 in TCGA and CGGA database indicated that SP1 levels were positively correlated with Glut1 and PKM2 (Fig. 5a, b). Previous studies suggest that SP1 activates Glut1 and PKM2 via binding motifs in promoters in cancers [23–25]. Therefore, we assumed that SP1 might regulate Glut1 and PKM2 expression at transcriptional level in GBM. To confirm this, we detected SP1, Glut1 and PKM2 levels in glioma tissues. As expected, we got similar results to analysis results of database (Fig. 5c). In Fig. 3c, we found that knockdown of SP1 significantly decreased Glut1 and PKM2 on protein levels in GBM cells. Here, we examined the mRNA level changes of Glut1 and PKM2 after SP1 knockdown. The results showed that knockdown of SP1 also significantly decreased Glut1 and PKM2 on mRNA levels in U87 and U251 cells (Fig. 5d). Then, we employed ChIP analysis to further validate the binding of SP1 to predicted binding regions of Glut1 and PKM2 (Fig. 5e). PCR products corresponding to the Glut1 and PKM2 promoter region containing specific binding motifs of SP1 were detected in U87 and U251 cells after SP1 immunoprecipitation (Fig. 5f). Next, to further examine whether SP1 could regulate Glut1 and PKM2 transcription activity via binding in the promoter of Glut1 and PKM2, we constructed wild type and mutant luciferase reporter (Fig. 5g). Luciferase reporter assays showed that the pGL3-WT reporter, rather than the pGL3-Mut reporter, was greatly activated by the overexpression of SP1 (Fig. 5h, i). These results confirmed the direct binding of SP1 to the promoter region of both Glut1 and PKM2.

### MiR-181b suppresses the tumorogenesis and progression by targeting SP1 in vivo

**Fig. 6:**
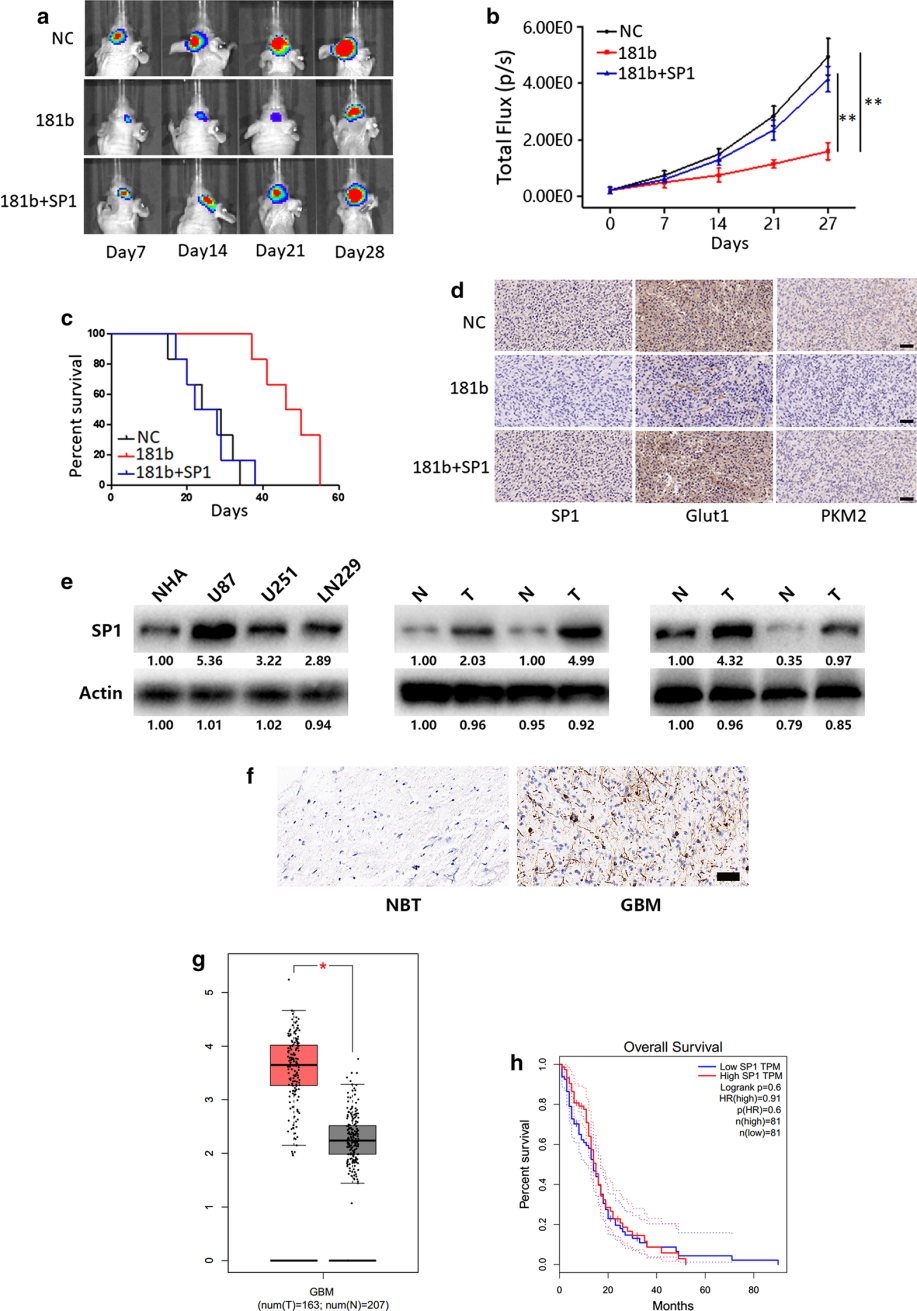
miR-181b overexpression inhibits glioma growth in vivo by targeting SP1. **a** Representative bioluminescence images of mice bearing U87 cells transfected with miR-NC, miR-181b or miR-181b plus SP1 on the days indicated (n = 10 each group). **b** Bioluminescence was quantified in tumors from three groups. **c** Kaplan–Meier survival curve of mice from three groups. **d** The expression of SP1, Glut1 and PKM2 were examined by Immunohistochemical staining of sections from a glioma xenograft model in mice. Scale bar = 50 μm. **e** The expression of SP1 were measured by Western blot in NHA, GBM cell lines, normal brain tissues (N) and tumors (T). **f** The expression of SP1 were examined by Immunohistochemical staining of sections from clinical normal brain tissues (NBT) and GBM samples. Scale bar = 50 μm. **g** SP1 expression was analyzed using TCGA data sets. **h** Kaplan–Meier survival curve of GBM patients with high or low SP1 levels according to TCGA data sets

Next, we tend to investigate whether miR-181b targeting SP1 could affect the tumorogenesis and progression in vivo. Luciferase-labeled U87/miR-NC or miR-181b was transfected with Luciferase. Meanwhile, we co-transfected SP1 in luciferase-labeled U87 cells overexpressing miR-181b. Then, cells were intracranially injected into female nude mice. Luminescence images were taken on day 7, 14, 21 and 28 to evaluate the growth of tumors. As shown in Fig. 6a, b, miR-181b overexpression displayed a significant reduction of tumor growth. Compared with miR-181b overexpression group, miR-181b plus SP1 overexpression could significantly promote the growth of tumors. The results were further confirmed by the survival curves (Fig. 6c). Histologic analysis indicated that tumors derived from U87/miR-181b cells had a significantly decreased SP1, Glut1 and PKM2 expression while miR-181b plus SP1 overexpression tumors expressed higher SP1, Glut1 and PKM2 levels than miR-181b overexpression tumors (Fig. 6d). Together, these results suggest that miR-181b could inhibit glioma growth via targeting SP1 in vivo.

Above results have verified miR-181b levels in clinical samples. As SP1 was directly regulated by miR-181b, we then analyzed SP1 levels in GBM cells and tissues. As shown in Fig. 6e, SP1 level was significantly higher in GBM cell lines (U87, U251, LN229) than in Normal human astrocyte; And SP1 levels were higher in tumors (T) than adjacent normal brain tissues (N). Meanwhile, IHC also suggested that SP1 was overexpressed in GBM tissues (Fig. 6f). Using GEPIA analysis tool, we identified that SP1 level was also higher in GBM than in normal brain tissues (Fig. 6g). However, there seems to be no survival benefit according to TCGA database (Fig. 6h). More clinical data are required for further analysis.

### Curcumin inhibits glioma cells aerobic glycolysis and proliferation via upregulating miR-181b

**Fig. 7:**
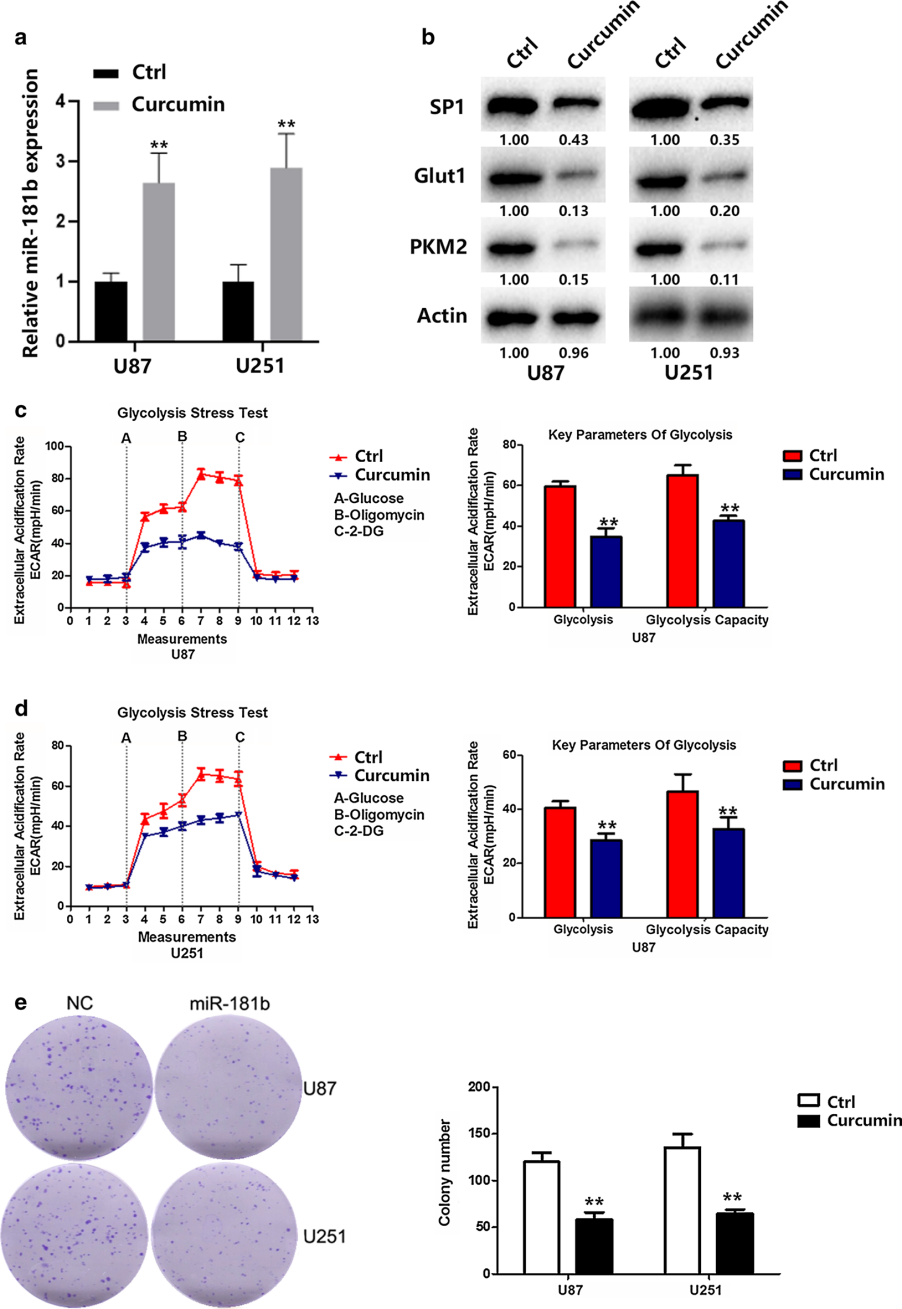
Curcumin inhibits glioma cells aerobic glycolysis and proliferation via upregulating miR-181b. **a** The expression of miR-181b were measured by qRT-PCR in U87 and U251 cells after the cells were treated with Curcumin. **b** The expression of SP1, PKM2 and Glut1 were measured by Western blot in U87 and U251 cells after the cells were treated with Curcumin. **c**, **d** ECAR was measured through the Glycolysis Stress in U87 and U251 cells after the cells were treated with Curcumin. **e** Colony formation ability of U87 and U251 cells after the cells were treated with Curcumin. *P < 0.05, **P < 0.01. All results are representative of at least three independent experiments

Since our results have identified the significance of miR-181b in GBM cells, we then tend to apply these information for GBM treatment. A large number of studies showed that natural compounds can modulate the expression of miRNA. Previous study suggested that Curcumin inhibited proliferation and induced apoptosis through upregulation of miR-181b in MDA-MB-231 breast cancer cells [26]. Therefore, we treated GBM cells with Curcumin. qRT-PCR results showed that Curcumin treatment significantly increased miR-181b levels in GBM cells (Fig. 7a). Western blot results showed that Curcumin treatment significantly decreased SP1, glut1, and PKM2 levels in GBM cells (Fig. 7b). In addition, glycolysis rate and glycolytic capacity of U87 and U251 cells were significantly reduced with Curcumin treatment (Fig. 7c, d). Meanwhile, Curcumin also inhibited the cell proliferation of U87 and U251 (Fig. 7e). Together, these results provided strong evidence for the anti-tumorogenesis role of miR-181b in GBM cells; and provide new strategy for GBM treatment.

## Discussion

Extremely activated metabolism is one of the features of malignant brain tumors [27]. Recently, studies have proved the significantly important role of miRNAs on regulating GBM cells glucose metabolism [28, 29]. MicroRNAs (miRNAs) are small non-coding, single-stranded RNAs, about 20–24 nt, which could affect the stability and translation of mRNAs by binding to the 3′UTR [30, 31]. Several studies have reported that miR-181 functions as a biomarker and has important roles in mass kinds of cancers, including glioma [32–34]. Xie et al. found that miR-181b could inhibits glycolysis in gastric cancer cells by targeting hexokinase 2 [35]. Xiao et al. revealed the role of miR-181b/HK2 axis on regulating aerobic glycolysis in prostate cancer [36]. Similarly, as shown in Fig. 1a, we found that overexpression could significantly inhibit glycolysis in glioma. In this study, we demonstrated that miR-181b is a critical regulator for the metabolic reprogramming of glioma cells for the first time. Enforced expression of miR-181b in GBM cells suppresses glycolytic activity and tumor growth in vivo.

Specificity protein 1 (SP1) is one of the most well characterized transcriptional activators [37]. SP1 and other family members are proved overexpressed in kinds of cancers. SP1 plays an important role in the regulation of cell metabolism and cell proliferation, including prominent oncogenes or tumor suppressors [38]. Using prediction website, we proposed that SP1 is the potential target of miR-181b. Then, luciferase activity assays and western blot verified our expectation. The rescue experiments also verified the results. Furthermore, we found positive relationship between SP1 and Glut1 or PKM2, which are important regulators of glycolysis [39–41]. As we all know, SP1 could bind to specific sequences which were involved in the expression and regulation of various genes [42]. Our research proved that SP1 could bind at specific motifs in PKM2 and Glut1 promoters confirmed by ChIP assays. Together, we proposed that dysregulation of SP1 by miR-181b could transcriptionally induce PKM2 and Glut1, and regulate glucose metabolism in glioma.

## Conclusion

In summary, our study certificated that miR-181b functioned as a tumor suppressor by directly targeting SP1 to prevent glucose metabolism and cell proliferation. We also demonstrated that miR-181b targeting SP1 could induce PKM2 and Glut1. Our results provide more evidence about miR-181b regulating glioma progression. Together with our previous results, our study indicates that miR-181b is a potential therapeutic target for GBM treatment.

## Data Availability

All data and material are available from the corresponding author.
